# Carbohydrate-insulin model: does the conventional view of obesity reverse cause and effect?

**DOI:** 10.1098/rstb.2022.0211

**Published:** 2023-10-23

**Authors:** David S. Ludwig

**Affiliations:** New Balance Foundation Obesity Prevention Center, Boston Children's Hospital, Boston, MA 02115, USA

**Keywords:** obesity, carbohydrate, insulin, diet, macronutrients, scientific models

## Abstract

Conventional obesity treatment, based on the First Law of Thermodynamics, assumes that excess body fat gain is driven by overeating, and that all calories are metabolically alike in this regard. Hence, to lose weight one must ultimately eat less and move more. However, this prescription rarely succeeds over the long term, in part because calorie restriction elicits predictable biological responses that oppose ongoing weight loss. The carbohydrate-insulin model posits the opposite causal direction: *overeating doesn't drive body fat increase; instead, the process of storing excess fat drives overeating*. A diet high in rapidly digestible carbohydrates raises the insulin-to-glucagon ratio, shifting energy partitioning towards storage in adipose, leaving fewer calories for metabolically active and fuel sensing tissues. Consequently, hunger increases, and metabolic rate slows in the body's attempt to conserve energy. A small shift in substrate partitioning though this mechanism could account for the slow but progressive weight gain characteristic of common forms of obesity. From this perspective, the conventional calorie-restricted, low-fat diet amounts to symptomatic treatment, failing to target the underlying predisposition towards excess fat deposition. A dietary strategy to lower insulin secretion may increase the effectiveness of long-term weight management and chronic disease prevention.

This article is part of a discussion meeting issue ‘Causes of obesity: theories, conjectures and evidence (Part II)’.

## 1. Introduction

***Note to Readers:** this paper is adapted from my oral presentation at the Royal Society's conference, Causes of obesity: theories, conjectures and evidence, session 3, 18 October 2022 (https://royalsociety.org/science-events-and-lectures/2022/10/causes-obesity/). Consistent with that presentation, this paper can be considered ‘The case for the carbohydrate-insulin model (CIM).’ The paper does not aim to provide, nor would length constraints allow, an exhaustive literature review or a balance of alternative views. Elsewhere, the reader can find comprehensive reviews by CIM proponents [1–3] and numerous opposing papers by critics [4–18].*

The First Law of Thermodynamics is often invoked to explain the pathophysiology of obesity. For instance, a 2017 scientific statement of the Endocrine Society (United States) concluded that ‘Obesity pathogenesis involves … sustained positive energy balance (energy intake > energy expenditure)’ [15, p. 268]. An expert panel of multiple professional organizations asserted in 2013, ‘To achieve weight loss, an energy deficit is required’ [19, p. S74]. However, equating a positive energy balance with increased adipose tissue mass, the body's primary energy storage depot, is a tautology, restating what we already know. The law of energy conservation dictates their equivalence:

$$\mathrm{positive}\;\mathrm{energy}\;\mathrm{balance}\;(E_{\mathrm{In}}\text{–}E_{\mathrm{out}})=\mathrm{increased}\;\mathrm{energy}\;\mathrm{storage}\;(\mathrm{adipose}\;\mathrm{mass})\;$$

However, this fact of physics does not inform pathogenesis. It is like saying the only way to make money in the stock market is to *buy low and sell high—*an observation that, while true, is meaningless as an investment strategy (electronic supplementary material, table S1 in reference [2] provides other examples of this tautology.).

For an informative translation of the First Law, one must replace the equal sign in the equation above with directional arrows. Does the positive energy balance drive excessive body fat storage? Or, conversely, does excessive deposition of body fat drive the positive energy balance? Neither possibility violates the laws of physics, but they have radically different implications to understanding the mechanistic basis of, and the treatment for, obesity.

## 2. Energy balance model—overeating drives increased adiposity

**Figure 1.**
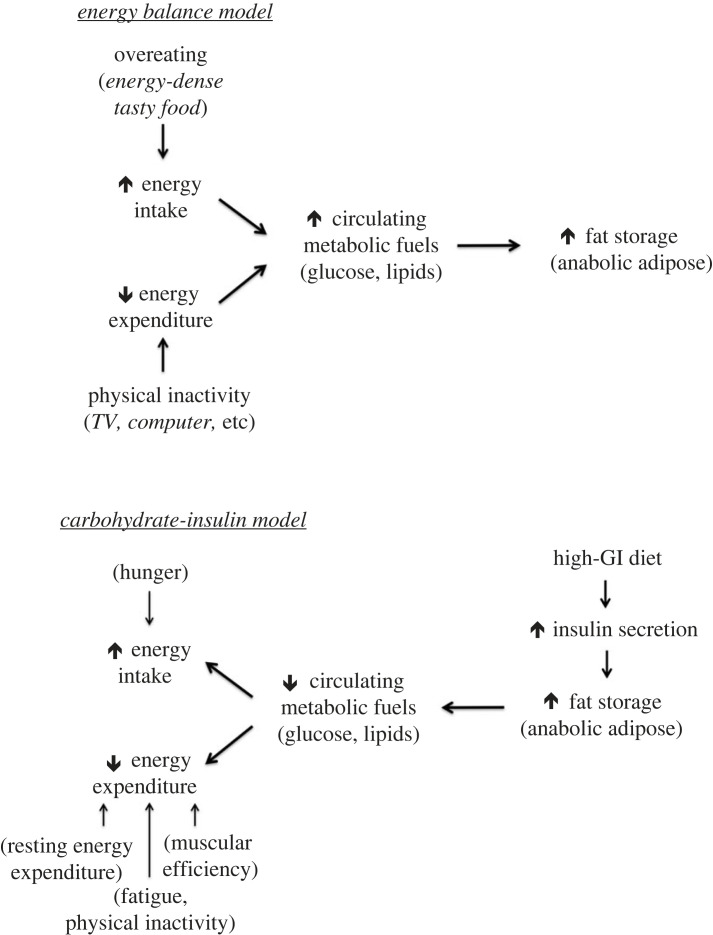
Opposing causal direction in obesity pathogenesis models. GI, glycemic index.

The conventional view, termed the energy balance model (EBM), considers overeating (*E*_In_ > *E*_Out_) the primary cause of obesity, as depicted in figure 1, top. In our modern food environment, it is easy to over consume energy-dense tasty foods and hard to burn off those extra calories amid our sedentary lifestyles. Consequently, calorie-rich substances build up in the blood stream and become deposited primarily into adipose tissue, and we gain weight.

Arguably, a fundamental assumption of the EBM, as recently underscored by several notable proponents, is that all calories are metabolically alike to the body. For instance, the Endocrine Society scientific statement asserts that, ‘The impact of diet on obesity risk is explained largely by its effect on calorie intake, rather than by changes of either energy expenditure or the internal metabolic environment. Stated differently, ‘a calorie is a calorie’’ [15, p. 268]. Other proponents stated ‘for all practical purposes, ‘a calorie is a calorie’ when it comes to body fat and energy expenditure differences between controlled isocaloric diets varying in the ratio of carbohydrate to fat’ [10, p. 1721].

Although recent formulations of the EBM have highlighted calorie-independent effects of food on the brain [4]—a notion that has not been much in dispute—these are proposed to act exclusively on pathways related to energy intake (e.g. satiety, reward) rather than energy expenditure or metabolic fuel partitioning. Indeed, the metabolic similarity of calories with respect to body fat storage is intrinsic to, and inseparable from, the EBM. Were this not so, then one diet with 2000 calories could lead to weight gain whereas another with the same calorie level but different macronutrients could lead to weight loss. This possibility would undermine the foundation of calorie counting and the very notion of an individual ‘energy requirement.’

If all calories are metabolically alike—that is, macronutrients have no unique effects on fat storage—then the only way to lose weight is to eat fewer of them or burn more off with physical activity. For more than 100 years, this paradigm has informed obesity treatment, beginning in the early 1900s with the best-selling diet book, *Diet and Health: with Key to the Calories* by Lulu Hunt Peters; continuing with the low-fat diet of the late 1900s, as exemplified by the US Department of Agriculture's first Food Guide Pyramid; and extending to a new EBM formulation that focuses on ‘inexpensive, convenient, energy-dense, ultra-processed foods that are high in portion size, fat and sugar, and low in protein and fibre’ that act on subconscious pleasure and reward pathways [4, p. 1244].

## 3. Anomalies

However, several anomalies—observations that cannot be neatly reconciled—raise questions about the validity and usefulness of this dominant paradigm. One anomaly is the continuing increase in obesity prevalence, despite persistent public health efforts focused on energy balance. For a century, advice to *eat less and move more* has been the mainstay of obesity prevention and treatment; packaged foods list calorie content; and a low-fat diet, targeting the most energy-dense macronutrient, has been advocated for decades [20]. However, if overeating were the primary cause of obesity, why has the campaign to prevent it been so spectacularly unsuccessful?

A second anomaly is the rising mean body mass index (BMI) among genetically stable populations. An average man in the United States (US) weighs about 15 kg more today compared to the 1960s. If that man lost 15 kg on an energy-restricted diet, he would typically experience not only increased hunger, but also a reduction in energy expenditure (i.e. to a lower level than experienced at the same weight by his counterpart before the obesity epidemic) [21]. Why does weight loss to a previously normal level elicit metabolic pushback?

Third, the obesity epidemic is associated with a tiny average daily energy imbalance. To develop obesity by the age of 35 involves the storage of just 1 g of excess fat daily (10 kcal, less than the energy in a teaspoon of sugar). Mean population energy intake since the 1970s has increased by only 200 to 250 kcal daily (a glass of grape juice) [22–24]. Considering how strongly many people want to prevent or treat obesity, why is it so difficult to control energy balance by eating just a bit less or exercising just a bit more? After all, adults routinely resist various temptations, such as illicit drugs and sex, that activate conscious or subconscious pleasure and reward pathways.

Fourth, as previously reviewed [1,2], many of the individual factors associated with the energy content of diet—food availability, energy density, dietary fat, tastiness, and food processing *per se—*do not seem to have an important effect on body weight, independent of confounding factors. With what confidence can we assume that a combined focus on all of these, as specified in recent EBM formulations, would have meaningful efficacy?

Fifth, since 2000, obesity rates have risen markedly without evidence of increased energy intake according to two methods of assessment: individual dietary recalls and energy in the food supply [24]. Although these methods may be biased, a new study found that basal energy expenditure has declined in the US and Europe since the 1980s, providing a third line of evidence for this anomaly [25]. Weight gain requires a positive energy balance, and heavier bodies require more energy for maintenance and physical activity. How can these divergent trends, if real, be reconciled with the EBM?

## 4. Carbohydrate-insulin model—increasing adiposity drives overeating

Although the First Law of Thermodynamics is not wrong, conventional assumptions about causal direction could be. Perhaps causality does not originate with the behavioural component of the pathway on the left in figure 1, top; but rather with the metabolic component on the right in figure 1, bottom. (For additional contrasts in the pathophysiological features of the EBM versus CIM, the reader is directed to table 1 in [1].)

In the CIM [1–3,26], a shift in substrate partitioning towards deposition rather than oxidation deprives metabolically active organs of available energy, most notably in the late postprandial state. Fuel sensing areas in the brain and perhaps liver, recognizing this metabolic state as a threat to energy homeostasis, respond by activating mechanisms that conserve and replenish available metabolic fuels—either by increased energy intake (via hunger and food reward pathways) or decreased energy expenditure (e.g. reduced resting energy expenditure, fatigue leading to less physical activity, increased muscular efficiency). Either way, a positive energy balance results from, rather than causes, increasing adiposity. Thus, the concentration of metabolic fuels in the blood, as a proxy for fuel sensing and oxidation, constitutes the regulated variable in the CIM, in contrast to body weight or fat mass in the EBM. A small shift of substrate partitioning towards fat deposition—a daily average of just 1 to 2 g extra—could account for the slow, progressive weight gain as seen in common forms of obesity.

What factors might induce this shift in substrate partitioning? That is ‘Endocrinology 101’—the insulin-to-glucagon ratio. Insulin is the dominant meal-influenced hormone, affecting the availability of all major metabolic fuels. Insulin stimulates the deposition of energy into adipocytes and inhibits lipolysis. States of high insulin action are associated with weight (fat) gain, such as exogenous insulin for treatment of diabetes, genetic variants that increase insulin secretion; and states of low insulin action are associated with weight (fat) loss, such as under-treatment with insulin for type 1 diabetes. Conversely, glucagon opposes these actions through catabolic effects [1,2].

What factors might have increased the insulin-to-glucagon ratio on a population basis, concomitant with the obesity epidemic? That is ‘Nutrition 101’—highly processed, rapidly digestible carbohydrates which increase insulin and suppress glucagon [27]. Consumption of these high-glycemic load (GL) foods—especially refined grains, potato products and added sugars—increased in absolute amount, and as a proportion of total energy intake, beginning in the 1970s as a consequence of the public health campaign to lower dietary fat [23,28–34]. The fructose component of sugar, although technically low-glycemic index (GI), may also drive fat deposition through unique metabolic actions in the liver [35].

## 5. Physiological mechanisms involving glycemic load

In a cross-over inpatient feeding trial [36], my collaborators and I fed 12 adolescents three breakfasts controlled for energy but differing in GL: (i) high-GL instant oatmeal (carbohydrate 64%, fat 20%, protein 16%); (ii) moderate-GL slow-cooking oatmeal (64/20/16); and (iii) low-GL vegetable omelette (40/30/30). After the breakfasts, we measured hormonal responses and metabolic fuel concentrations for 5 h. Subsequently, we fed the same meals for lunch and monitored ad libitum food intake. As expected, insulin rose higher after the high-GL breakfast than after moderate- or low-GL breakfasts. Simultaneously, levels of glucagon were suppressed following the high-GL breakfast. This highly anabolic hormonal response would promote uptake of glucose into liver and lipids into adipose tissue, restrain hepatic release of glucose, and suppress lipolysis. Consequently, the concentrations of glucose and non-esterified fatty acids were lowest after the high-GL meal, compared to the other meals, beginning approximately 2.5 h postprandially. A surge in the counter-regulatory hormone epinephrine after approximately 4 h highlights the potential biological significance to the brain of these metabolic events. With free access to food, participants consumed 600 to 700 kcal more following the high-GL lunch, a finding consistent with most other short term trials [37]. However, diet effects on energy intake from these short trials must be interpreted cautiously, as considered below.

Shifting emphasis to the other component of energy balance, my collaborators and I examined the effects of GL on total energy expenditure (TEE) in a five month outpatient feeding trial [38]. Following approximately 10% weight loss on an energy-restricted run-in diet, 164 adults were randomly assigned to three weight-maintenance diets using a parallel design: low-carbohydrate (20%), moderate-carbohydrate (40%) or high-carbohydrate (60%), controlling protein at 20%. Total dietary energy was periodically adjusted to keep weight ±2 kg of the post-weight loss level. As in the crossover trial above, we observed a decline in circulating metabolic fuels in the late postprandial phase on the high-carbohydrate diet (a difference among diets of about 0.5 kcal l^−1^) [39].

TEE decreased on the high-carbohydrate diet, remained the same on the moderate-carbohydrate diet, and increased on the low-carbohydrate diet, a difference of approximately 200 to 250 kcal d^−1^ in the intention-to-treat and per protocol models [38]. Regarding internal and external validity, this dietary effect: (i) remained robust in reanalyses accounting for plausible dietary non-adherence; (ii) corresponded closely to the observed energy requirements necessary to achieve weight maintenance in the three diet groups; and (iii) is consistent with a recent meta-analysis of trials lasting greater than two weeks [40–43]. A novel mechanism contributing to this difference in TEE may be suppression of adipose mitochondrial respiration on a high-carbohydrate diet [44].

These findings challenge critical aspects of EBM-based obesity treatment. If two isocaloric diets could have opposite effects on weight arising from differences in metabolism, then calorie-counting to achieve a negative energy balance—indeed, the very notion of an intrinsic energy requirement—may lack biological foundation.

## 6. Chronic effects of glycemic load in animals

Virtually all long-term human trials have major methodological issues limiting generalizability, related to the difficulty of controlling all dietary and behavioural factors that might confound clinical outcomes. For this reason, laboratory animal research has been employed to examine predictions of obesity models.

**Figure 2.**
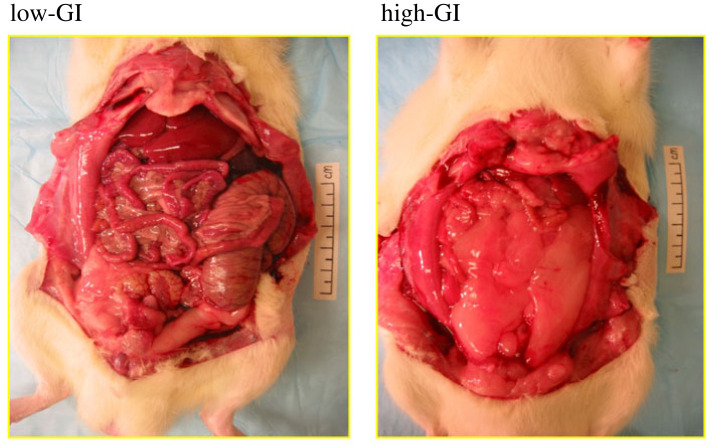
High-glycemic index (GI) diet rodent obesity model. Representative weight-matched animals consumed diets with low-GI versus high-GI, as described in Pawlak *et al*. [46]. Despite consuming less energy, the high-GI animal had greater fat mass and less lean mass—prima facie evidence for a defect in substrate partitioning. Adapted from Ludwig *et al*. [45].

In one study from a long line of investigation involving numerous research laboratories (reviewed in [45]), my collaborators and I gave Sprague-Dawley rats macronutrient-controlled high- versus low-GI diets, using a feeding protocol to maintain similar mean body weight between groups [46]. Initially, both groups of animals ate ad libitum and had similar weight gain. However, at seven weeks, the high-GI animals began to gain more weight than the low-GI group without an evident difference in food intake, suggestive of decreased TEE. Therefore, to maintain weight parity, we restricted food for the high-GI animals. At 18 weeks, the high- versus low-GI groups had virtually identical mean body weight (548 versus 549 g), but the former had a 71% increase in body fat, and a commensurate reduction in lean body mass. Figure 2 shows representative animals with the same weight. The one from the low-GI group ate ad libitum and had greater lean mass whereas the one from the high-GI group *consumed fewer calories* and yet had *greater fat mass*. These findings cannot be explained by the conventional energy balance view and instead comprise a prima facie case for a disorder in substrate partitioning. Furthermore, features of this model demonstrate the state of ‘internal starvation’ coexisting with excessive adiposity as postulated in the CIM, including increased hunger, reduced TEE, reduced lean mass and insulin resistance.

These metabolic effects seem more the rule than the exception in animal models of obesity. In a recent review, my coauthors and I characterized mechanistic features of these models (table 2 in [1]). In addition to the high-GI diet model, animals with melanocortin 3 (MC3) receptor deficiency manifest increased adiposity without increased weight, indicating the presence of a causal pathway consistent with the CIM. In a second group of models (including Agouti-related protein neuron ablation, gamma-aminobutyric acid (GABA) deficiency, melanocortin 2 receptor accessory protein 2 deficiency and a high-sugar diet), increased adiposity develops before increased food intake, supporting the CIM. In a third group (leptin deficiency, melanin concentration hormone excess, MC4 receptor deficiency, neuropeptide Y excess), increased adiposity occurs with control of food intake, such as with pair-feeding, consistent with the CIM. Thus, increased food intake may be necessary for obesity phenotypes to manifest fully, but disorders in substrate partitioning can often be observed without increased energy intake. These examples also demonstrate the calorie-independent metabolic effects of so-called ‘hunger’ and ‘satiety’ hormones. Contrary to a common assumption, the presence of these hormones in brain areas affecting food intake does not necessarily imply that they act directly and exclusively through increasing energy intake.

## 7. Chronic effects of glycemic load in humans

Short-term macronutrient trials characteristically show an adverse effect of low-carbohydrate diets on metabolism or body weight. For example, among 20 adults with high BMI, ad libitum energy intake was 689 kcal d^−1^ greater on a very-low-carbohydrate diet versus a low-fat diet during two week trial arms [47]. However, this diet effect had already diminished markedly during the second week, by about 300 kcal d^−1^, suggesting it could extinguish after another week or two. Indeed, physiological studies dating back decades show that adaptation to a major change in macronutrients requires at least two to three weeks [40,48–51]. This transient adaptive process explains findings of a new meta-analysis [40]: comparing low-carbohydrate to low-fat diets, energy expenditure was slightly reduced in trials of less than two weeks but more substantially increased in longer trials.

Among more informative, long-term trials, meta-analyses indicate that low-fat diets are *inferior* to higher-fat/lower-carbohydrate diets for weight control, compared head-to-head [52–54]—contrary to prior EBM-based prediction [29–34]. Admittedly, these differences are not large, typically 1 to 2 kg, and the effectiveness of the low-carbohydrate diet in these trials wanes with time. However, most of these trials involve weak behavioural interventions that fail to maintain meaningful dietary differentiation between treatment groups. It remains unclear whether these small effect sizes reflect limited inherent efficacy or poor study implementation. Distinguishing among these possibilities requires more powerful intervention, such as with at least partial food provision and strong participant supports. Among the relatively few trials that use intensive interventions, larger and sustained advantages for the low-GL diet have been observed [55]. In Diogenes, diets with progressive decreases in GL led to progressive improvement in weight loss maintenance [56]. An important methodological limitation with all these studies is potential confounding by related or compensatory dietary components. Some low- versus high-GI foods tend to be higher in fibre, resistant starch, and microntrients, for example, and these could mediate or otherwise influence some of the observed diet effects. Furthermore, with a decrease in one dietary factor, other factors will probably increase, leaving uncertainty about proximate drivers.

## 8. Evidence from pharmacology

As previously reviewed [1], drugs that increase insulin secretion or insulin action at adipose tissue are associated with weight gain, including insulin itself, sulphonylureas, and the thiazolidinediones. Conversely, drugs that decrease insulin secretion characteristically cause weight loss, such as alpha-glucosidase inhibitors, diazoxide and sodium-glucose cotransporter 2 inhibitors.

Some argue that the marked weight loss with new glucagon-like peptide 1 (GLP-1) receptor agonists cannot be reconciled with the CIM, because this drug class increases insulin secretion (see reference [4] and the live question and answer period following my talk, *ca* minute 33:00 https://www.youtube.com/watch?v=hkuktx5FUto). However, this view of GLP-1 receptor agonists and insulin secretion is not generally correct (i.e. other than in the context of severe β-cell failure) [57]. Whereas GLP-1 directly simulates β-cell insulin secretion, both GLP-1 and the long-acting agonists decrease insulin levels in response to a mixed meal—the clinically relevant context. Acutely, GLP-1 delays gastric emptying and slows nutrient absorption (lowering the GI of a meal in effect), and these actions dominate insulinotropism, as repeatedly demonstrated in clinical trials [58–65]. Chronically, the effects of long acting agonists on gastric emptying wane, although these do not dissipate entirely [66,67], a controversy complicated by methodological issues in measuring the relevant phase of gastric emptying [68,69]. Moreover, the effects of GLP-1 receptor agonists on gastric emptying predict long-term weight loss [67]. These drugs also lower insulin secretion by improving β-cell sensitivity to glucose.

Related to this issue, the physiological actions of GLP-1 produced in the gastrointestinal tract are clearly peripheral, as the half-life in circulation of this hormone is too short for a biologically meaningful amount to enter the brain. Long-acting agonists have major actions in the central nervous system, but these pharmacological mechanisms do not inform this debate. All models of obesity pathogenesis must allow for the presence of brain centres that control eating behaviour. The central site of action does not necessarily mean that the drug acts directly and exclusively on food intake, independent of a feedback loop involving peripheral metabolic mechanisms. In animals, GLP-1 receptor agonists have calorie-independent metabolic actions involving the autonomic nervous system that affect substrate partitioning [70,71]. The extent to which these may be present in humans remains unclear.

## 9. Carbohydrate-insulin model as a framework for other dietary and non-dietary exposures

I have argued that the increased dietary GL on a population basis since the low-fat diet era of the late twentieth century has raised secretion of insulin relative to glucagon, triggering excessive fat storage in adipose tissue. This shift in substrate partitioning, associated with decreased metabolic fuel availability, drives the positive energy balance that must accompany weight gain. Beyond a single dietary factor, this model provides a conceptual framework for understanding how other dietary factors (including fructose, protein, fatty acid type), the gut microbiome, physical activity, sleep, stress, and endocrine disrupting environmental exposures could cause obesity, not by primary actions on brain hunger centres, but at least partially through metabolic mechanisms [2].

## 10. Refining models of obesity pathogenesis

Ultimately, all models of complex biological phenomena are at best approximations—useful to the degree that they stimulate thinking and inform research. My collaborators and I recognize that the CIM: (i) lacks critical evidentiary support in some areas); (ii) will require revision as new data accrue; (iii) cannot explain all of the variance in BMI observed during the obesity epidemic; and (iv) does not exclude other explanatory models that operate along different causal pathways. More work will be needed to define the subset of individuals most susceptible to GL (i.e. precision nutrition) [72]; clarify mechanisms, such as those relating decreased metabolic fuel availability in the last postprandial period to hunger and energy expenditure; and refine behavioural interventions to facilitate public health translation.

Nevertheless, the CIM targets specific, modifiable environmental factors and proposes numerous specific, testable hypotheses. The same may not be true of the EBM. Commenting on its invocation of overly broad dietary factors and lack of specific mechanisms, Jeffrey Flier, former Dean of Harvard Medical School, recently wrote [73, pp. 3-4]:

[C]an we conclude there is an ‘‘energy balance model of obesity’’ or EBM – in the same sense that CIM is proposed to be such a model? I believe the answer to that question is no.… As a model to explain the rising prevalence of obesity, the EBM must do more than reference known components of this homeostatic system, including those that have proven incapable of preventing the rising prevalence…. [It should also] propose approaches to explain how specific environmental influences enable known anti-obesity homeostatic defenses to be evaded in some (but not all) members of the population.’

Thus, to promote constructive scientific debate, deficiencies of the EBM should be addressed in a new formulation.

## 11. Towards an integrated model

In conclusion, I would like to readdress a question posed at the beginning of my talk. *Which comes first, overeating or increasing adiposity?* Perhaps both.

**Figure 3.**
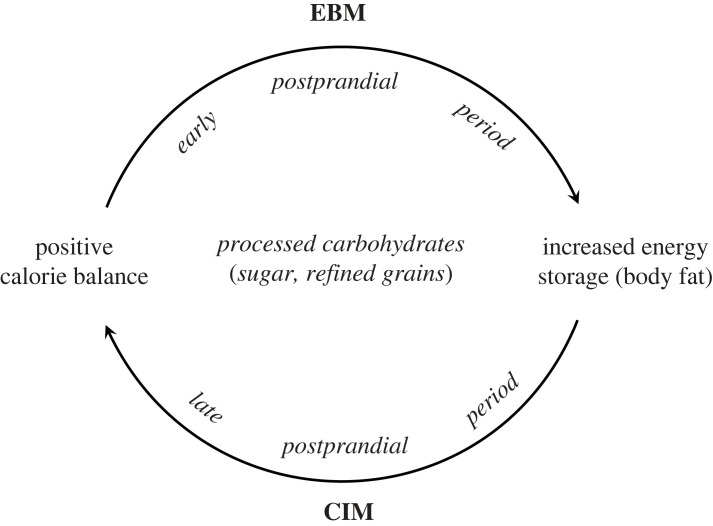
An integrated model of obesity pathogenesis with bi-directional causality. CIM, carbohydrate-insulin model; EBM, energy balance model.

As depicted in figure 3, hedonic and reward aspects of food could cause people to eat more than necessary to satisfy energy requirements, as postulated by the EBM (comprising a ‘push’ mechanism). Then, in the late postprandial phase, anabolic hormonal responses to the meal might trap those extra calories into adipose tissue, and suppress their release, as specified in the CIM (a ‘pull’ mechanism) contributing to a vicious cycle of overeating and weight gain. The relative contributions of these mechanisms may vary between people, with differing physiology (e.g. insulin secretion [72]), metabolic health state, and for other reasons, explaining some of the heterogeneity in response to different weight loss diets. Regarding public health implications, refined grain products and added sugars comprise an obvious target for intervention, identified by both models, albeit for different (but potentially synergistic) reasons. Thorkild Sørensen and I explore this integrated model in a recent commentary [74].

## Data Availability

This article has no additional data.
